# The Combination of Rituximab and Bendamustine as First-Line Treatment Is Highly Effective in the Eradicating Minimal Residual Disease in Follicular Lymphoma: An Italian Retrospective Study

**DOI:** 10.3389/fphar.2017.00413

**Published:** 2017-06-29

**Authors:** Sara Galimberti, Elena Ciabatti, Giacomo Ercolano, Susanna Grassi, Francesca Guerrini, Nadia Cecconi, Martina Rousseau, Giulia Cervetti, Francesco Mazziotta, Lorenzo Iovino, Franca Falzetti, Flavio Falcinelli, Alberto Bosi, Luigi Rigacci, Sofia Kovalchuk, Daniele Vallisa, Lucia Macchia, Eugenio Ciancia, Mario Petrini

**Affiliations:** ^1^Section of Hematology, Department of Clinical and Experimental Medicine, University of PisaPisa, Italy; ^2^Doctoral School of Genetics, Oncology and Clinical Medicine (GeNOMEC), University of SienaSiena, Italy; ^3^Department of Hematology, University of PerugiaPerugia, Italy; ^4^Department of Hematology, University of FirenzeFirenze, Italy; ^5^Department of Hematology and Oncology, Azienda Unità Sanitaria Locale di PiacenzaPiacenza, Italy; ^6^Pathology, Azienda Ospedaliero Universitaria PisanaPisa, Italy

**Keywords:** MRD, follicular lymphoma, bendamustine, BCL2/IGH, rituximab, PCR

## Abstract

R-Bendamustine is an effective treatment for follicular lymphoma (FL). Previous large trials demonstrated the prognostic role of the molecular minimal residual disease (MRD) during the most frequently adopted chemotherapeutic regimens, but there are not yet conclusive data about the effect of combination of rituximab (R) and bendamustine in terms of MRD clearance. Thus, the aim of this retrospective study was to assess if and in what extent the combination of rituximab and bendamustine would exert a significant reduction of the molecular disease in 48 previously untreated FL patients. The molecular marker at baseline was found in the 62.5% of cases; no significant differences were observed between patients with or without the molecular marker in respect of the main clinical features. Moreover, the quantization of the baseline molecular tumor burden showed a great variability: the median value was 1.4 × 10^−2^ copies, ranging from 3 × 10^−5^ to 4 × 10^4^. The initial molecular tumor burden did not correlate with clinical features and did not impact on the subsequent quality of response. After treatment, 93% of cases became MRD-negative; the median reduction of the *BCL2/JH* load was 4 logs. The 2-years PFS was 85%; it was significantly longer for patients in complete than for those in partial response (91 vs. 57%; *p* = 0.002), and for cases with lower FLIPI-2 score (88 vs. 60%; *p* = 0.004). On the contrary, PFS did not differ between patients with or without the molecular marker at baseline; a molecular tumor burden 15 times higher was observed in the relapsed subgroup in comparison to the relapse-free one, but this difference did not change the PFS length. The 2-years OS was 93.6%; the only variable that significantly impacted on it was the FLIPI-2 score; the presence of the molecular marker at baseline or its behavior after treatment did not impact on survival. This study, even if retrospective and conducted on a small series of patients, would represent a proof of concept that R-bendamustine is able to so efficaciously eradicate MRD that it could be able to by-pass the prognostic significance of MRD already demonstrated for other chemotherapeutic regimens in FL.

## Introduction

The follicular lymphoma (FL) is the second more frequent histotype of non-Hodgkin's lymphomas in the Western world, and represents one of the entities where the minimal residual disease (MRD) has been more frequently studied. Indeed, the history of MRD in FL is now 15 years old, starting with the demonstration that rituximab (R) significantly reduced the molecular tumor burden when administered after the CHOP (Cyclophosphamide, Doxorubicin, Vincristine, Prednisone) regimen: in that setting, patients achieving MRD negativity showed a significantly longer event-free survival (Rambaldi et al., 2002). Then, many other works focused on the MRD in the transplanted patients: cases MRD-negative after autologous transplantation remained relapse-free and alive in a higher percentage than those persistently MRD-positive (Galimberti et al., 2003; Melillo et al., 2005).

Subsequently, when yttrium-ibritumomab tiuxetan as consolidation was compared to the observation only, it was evident that radio-immunotherapy was able to rapidly eradicate MRD in the majority of patients (Goff et al., 2009): after this treatment 77% of patients became PCR-negative, with a significant favorable impact on progression-free survival (PFS) (Ibatici et al., 2014).

In the 2010, van Oers and colleagues demonstrated, in a series of relapsed patients, that the presence of the *BCL2/JH* at diagnosis had a negative impact on the outcome, whereas the molecular status before rituximab maintenance did not discriminate patients with worse outcome (van Oers et al., 2010).

More recently, two large studies conducted by the Fondazione Italiana Linfomi (FIL) showed that after R-CHOP, R-CVP (cyclophosphamide, vincristine, prednisone), R-FM (fludarabine and mitoxantrone) and R-FND (fludarabine, mitoxantrone, dexamethasone) the MRD status significantly conditioned the PFS; indeed, patients in partial response (PR) but MRD-negative showed longer PFS than those in complete remission (CR) but still MRD-positive (Ladetto et al., 2013; Galimberti et al., 2014).

More recently, the appearance in the scenario of the indolent lymphomas of the new anti-CD20 monoclonal antibody, obinutuzumab, prompted researchers to evaluate its value also in the context of the molecular response. In the Gallium trial, where CHOP, CVP or bendamustine were combined with rituximab or obinutuzumab, the new antibody resulted more powerful than rituximab, offering 92.5% of MRD-negativity when combined with bendamustine, 91.3% with CHOP, and 91.4% with CVP. Interestingly, the use of obinutuzumab in respect of rituximab added 13.5% of good molecular responses in the cohort receiving CHOP, 15.4% in those patients treated with CVP, but only 3% in the bendamustine arm, thus suggesting that molecular clearance offered by bendamustine was higher than that induced by the other two chemotherapeutic regimens (Pott et al., 2016).

Then, MRD is still today a hot topic and often more frequently it is introduced as aim in several phase-3 trials, as in the FIL FOLL12 study (trial.gov identifier number: NCT02063685), where the experimental arm is based on the PET and MRD results: a de-intensified treatment was reserved to MRD- and PET-negative cases, a consolidation with radio-immunotherapy to patients still PET-positive after induction or a pre-emptive therapy was adopted for PET-negative but MRD-positive cases.

Nevertheless, MRD is still not a decisional tool in the routine clinical practice: indeed, the ESMO guidelines recently edited, even if clearly recognizing the prognostic role of the MRD, state that the MRD “still cannot lead the therapeutic strategy” (Dreyling et al., 2017).

In this context, few data about R-bendamustine and MRD in FL are today available: Zohren and colleagues reported that 89.5% of cases receiving R-bendamustine achieved the MRD negativity, with a significant advantage in terms of PFS (Zohren et al., 2015).

More recently, in the context of Gadolin trial, obinutuzumab combined with bendamustine induced 82% of MRD negativity vs. 43% of the bendamustine alone, with a significant longer PFS for MRD-negative cases (Pott et al., 2015).

With these premises, we decided to assess the impact of the R-bendamustine on MRD in a cohort of 48 FL patients treated in 4 different Italian centers. The aim of this retrospective study was to measure both by qualitative and quantitative PCR the molecular tumor burden before and 2 months after the end of treatment with R-bendamustine. The presence and quantization of the *BCL2/JH* rearrangement at baseline and its behavior after induction was then compared with clinical features, response rates, and 2-years survivals.

## Patients and methods

### Patients

This retrospective observational study was conducted in 4 Italian centers (Pisa, Firenze, Perugia, and Piacenza), in the context of the routine clinical practice.

Patients with newly diagnosed FL received bendamustine 90 mg/m^2^, days 1 and 2, once a month, plus rituximab 375 mg/m^2^, for total 6 cycles.

All patients signed a consent for leaving the leftover of samples used for routine molecular analyses for further scientific purposes. Forty-eight patients affected by FL were enrolled into the study on the basis of the residual DNA availability; the *BCL2/JH* rearrangement was evaluated on samples harvested at diagnosis and 8–10 weeks after the end of induction.

Samples have been collected between January 2012 and December 2015.

In addition to the physical examination and imaging (total body computed tomography scan, ultrasonography or x-ray, according to physicians' decision), at diagnosis all patients underwent bone marrow (BM) biopsy and aspirate. In each center, BM biopsies were evaluated also by immunohistochemistry (at least CD20, CD10, CD5, kappa, and lambda clonality), in order to confirm the morphological diagnosis of FL.

Imaging was performed again for assessment of response at treatment completion in addition to physical examination and laboratory tests. Quality of response was defined according to the standardized international criteria (Cheson et al., 2016).

### Molecular assays

Qualitative *BCL2/IGH* rearrangement analysis was performed at baseline and at the end of treatment. All qualitative and quantitative analyses were centralized at the molecular laboratory of the Hematology of the Pisa University, Italy.

DNA was extracted from BM mononuclear cells by Wizard Genomic DNA purification kit (Promega Madison WI, USA), according to the suggestions by the FIL MRD Network (Mannu et al., 2015). In order to amplify *BCL2/IGH* rearrangement, nested PCR reactions were performed as previously described, both for the MBR and mcr breakpoints (Galimberti et al., 2014).

The sensitivity of the PCR assays was confirmed by testing serial dilutions of DNA derived from the *BCL2/IGH*-positive DOHH-2 cell line, achieving a limiting dilution of 1:10^−5^, either for qualitative or quantitative tests.

### Statistical analysis

All statistical analyses were performed using the SPSS 21.0 software (SPSS Inc., Chicago, IL, USA). OS was calculated from the date of diagnosis to death or last follow-up; PFS was measured from the date of response to the induction treatment to the last follow-up, disease progression or relapse. Survival curves were calculated using the Kaplan-Meier method, and statistical comparisons between curves were made using the log-rank test. *Post-hoc* comparisons were obtained using the Cox proportional hazard regression method. The Chi squared test, Fisher's exact test and Kruskal-Wallis test were used to compare variables when appropriate. For establishing a possible value of quantitative *BCL2/IGH* rearrangement on relapse, a ROC curve was also performed. All statistical comparisons were two-sided. The date of the last molecular follow-up was October 2016.

## Results

### Baseline: patients characteristics and molecular marker

Characteristics of the 48 patients enclosed into the study are reported in the Table 1.

**Table 1:** Clinical characteristics of the enrolled patients.

**Characteristics**	**Number (%)**
Number of patients	48
Median age, years	63
range	36-83
**SEX**	
Male	24 (50%)
Female	24 (50%)
**HISTOTYPE**	
Grade 1	17 (35%)
Grade 2	24 (50%)
Grade 3	7 (15%)
**ANN ARBOR STAGE**	
II	8 (16%)
III	20 (42%)
IV	20 (42%)
ECOCG Performance Status >1	4 (8%)
**FLIPI2**	
Low	9 (19%)
Intermediate	31 (65%)
High	8 (16%)
BM involvement	20 (42%)

Their median age was 63 years (range 36–83), and half of them were male.

By microscope observation, BM resulted infiltrated in 20 cases; the 85% of them were scored as showing a grade 1 or 2 FL; 16% of our patients were in stage II, 42% in stage IV, and more than 60% presented with intermediate FLIPI-2 risk score.

By qualitative PCR, *BCL2/JH* rearrangement at baseline was found in 30 patients (62.5%). The breakpoint was MBR in 28 cases, and mcr in the remaining two.

In 3 out of the 20 cases with microscopic BM infiltration (15%) we did not find the *BCL2/IGH* rearrangement, probably for the presence of some rare breakpoints in the *BCL2* gene (not assessed in this study). On the other hand, in 9 of the 28 cases without BM infiltration (32%), the molecular marker was found, possibly due to a submicroscopic involvement.

In this study, the translocation between chromosome 14 and 18 (characteristic of FL) was not assessed.

At baseline, the molecular tumor burden was also assessed by quantitative PCR (QT-PCR) in the 28 cases with the MBR breakpoint; it was quantified in 27 of them, because in one case the *BCL2/JH* rearrangement resulted at the lowest limit of the test sensitivity, and thus positive but not quantifiable. As expected, measures showed a wide inter-patients variability: the median value was 14 × 10^−2^ copies, but it ranged from 3 × 10^−5^ to 4 × 10^4^ copies.

No significant differences were observed for the main clinical features (age, sex, BM, infiltration, stage, histological grade, FLIPI-2 score) between patients with or without molecular marker detected by qualitative or quantitative PCR (Table 2).

**Table 2:** Comparison of the patients' clinical characteristics according to the presence/absence of the molecular marker at baseline.

**Characteristics**	**Patients with molecular marker number (%)**	**Patients without molecular marker number (%)**	**p**
Number of patients	30	18	
Median age, years	61	64	
range	40-83	36-79	n.s.
**SEX**			
Male	16 (53%)	8 (45%)	
Female	14 (47%)	10 (55%)	n.s.
**HISTOTYPE**			
Grade 1	11 (37%)	6 (33%)	n.s.
Grade 2	15 (50%)	9 (50%)	
Grade 3	4 (13%)	3 (17%)	
**ANN ARBOR STAGE**			
II	5 (17%)	3 (17%)	n.s.
III	12 (40%)	8 (44%)	
IV	13 (43%)	7 (39%)	
ECOCG Performance Status >1	3 (10%)	1 (6%)	n.s.
**FLIPI2**			
Low	6 (20%)	3 (17%)	n.s.
Intermediate	19 (63%)	12 (67%)	
High	5 (17%)	3 (17%)	
BM involvement	12 (40%)	8 (44%)	n.s.

### Clinical response to treatment

All 48 patients responded to treatment, 38 of them (79%) achieving a complete response (CR).

The achievement of CR was more frequently observed in cases with low/intermediate than in those with high FLIPI-2 score (87 vs. 20%; *p* = 0.004).

No other clinical features impacted on response rate or on the quality of response.

In particular, the presence of the molecular marker at baseline did not correlate with the subsequent quality of response to treatment: indeed, the CR rate was 45% in the subgroup of cases with *BCL2/JH* detectable at baseline vs. 55% in the PCR-negative cohort (*p* = 0.49).

Also the molecular tumor burden (according to the median value or the 1 or 4th quartile) did not impact on the response rate.

### Overall and progression-free survival

**Figure 1:**
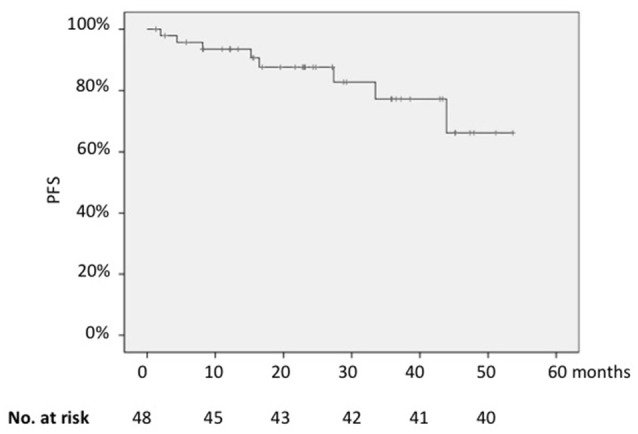
Kaplan-Meier analysis of progression-free survival of the whole cohort.

In the whole series, the 2-years PFS was 85% (Figure 1); it was significantly longer for patients in CR than for those in partial remission (PR) (91 vs. 57%; *p* = 0.002) and for cases with low vs. those with high FLIPI-2 score (88 vs. 60%; *p* = 0.004).

On the contrary, PFS did not differ between patients with or without the molecular marker at baseline when *BCL2/JH* rearrangement was assessed by qualitative PCR (2-years PFS, 83% for patients *BCL2/JH*-positive vs. 91% for those *BCL2/JH*-negative; *p* = 0.25).

**Figure 2:**
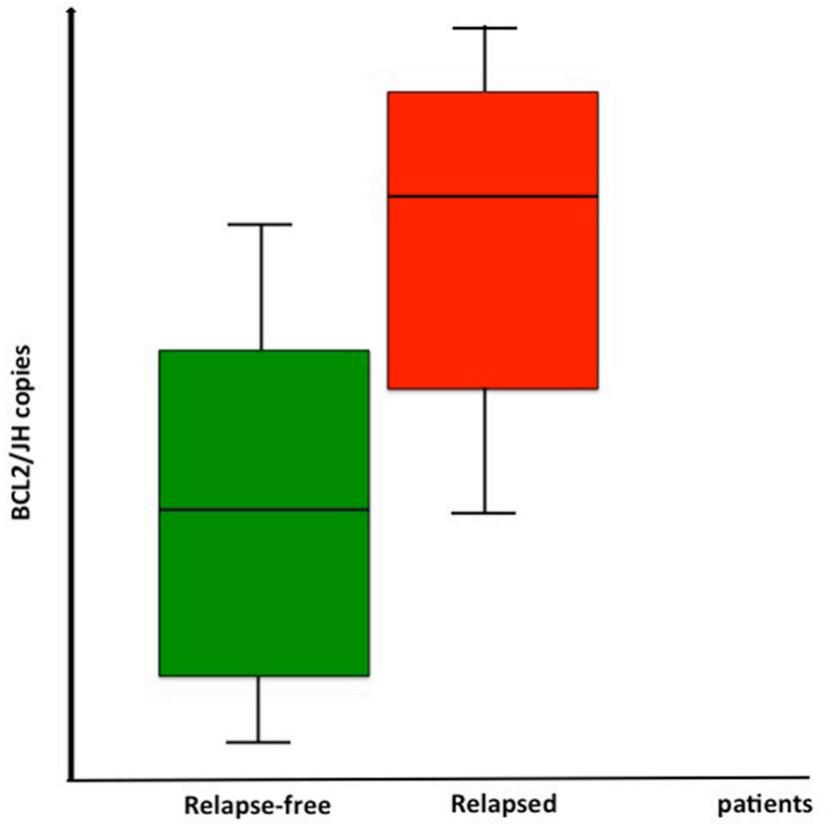
Correlation between molecular tumor burden at baseline and relapse rate: higher initial molecular burden was significantly correlated with a higher probability of relapse/progression (mean of *BCL2/JH* copies in the relapse-free subgroup: 9,411.8 ± 17,489.5 vs. 133,333.1 ± 230,940.1 in the relapsed cases; *p* = 0.022).

At the contrary, a higher initial molecular burden was significantly correlated with a higher probability of relapse/progression (mean of *BCL2/JH* copies in the relapse-free subgroup: 9,411.8 ± 17,489.5 vs. 133,333.1 ± 230,940.1 in the relapsed cases; *p* = 0.022; Figure 2).

Nevertheless, when we stratified patients according to the median number of *BCL2/JH* copies (0.014) or according to quartiles (75 or 25th percentile), no differences in terms of PFS were observed (2-years PFS, 100% for cases with *BCL2/JH* ratio < 0.014 vs. 81% of cases with higher molecular tumor burden; *p* = 0.08).

When the PCR status at baseline, quality of response (CR vs. PR) and FLIPI-2 score (high/intermediate vs. low) were inserted as covariates in the multivariate analysis, all parameters lost their statistical significance.

**Figure 3:**
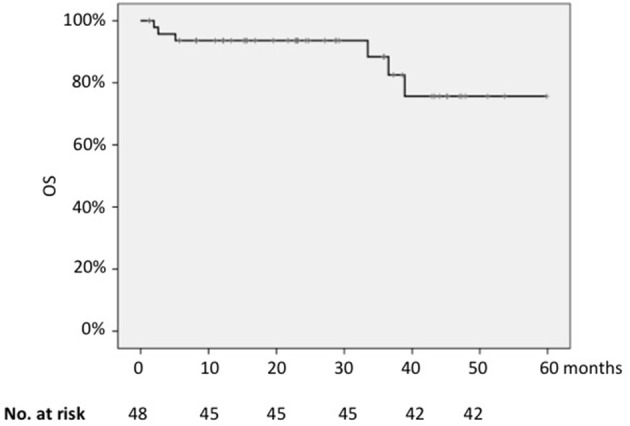
Kaplan-Meier analysis of overall survival of the whole cohort.

The 2-years OS for the whole series was 93.6% (Figure 3); the only parameter that significantly impacted on survival was the FLIPI-2 score, with 2-years OS of 97% for cases with low FLIPI-2 vs. 60% for those with high risk score (*p* = 0.001).

Differently from the PFS, the quality of response was not prognostic in terms of OS (2-years OS 57% for patients in CR vs. 37% for those in PR; *p* = 0.55).

Concerning the value of the presence of the molecular marker or its load at baseline, the PCR status before treatment did not significantly impact on OS.

### The impact of treatment on MRD

After induction, all initially PCR-positive patients were re-assessed by both qualitative and quantitative PCR: the qualitative analysis became negative in all cases except two (93.3%); also in the two patients with the mcr breakpoint, the molecular marker disappeared.

In addition, QT-PCR showed the achievement of a very deep response in the majority of cases: 25 patients (92.6%) achieved the MRD negativity (≤ 1 × 10^−6^ copies), and 2 resulted positive but not quantifiable (molecular tumor burden between 1 × 10^−5^ and 1 × 10^−6^). Overall, the median reduction of *BCL2/JH* burden was 4 logs, with 6 cases showing a reduction around or higher than 5 logs.

No significant differences in term of probability of relapse or death nor in terms of PFS and OS length were observed in cases with quantitative reductions higher or lower than the median of 4 logs.

## Discussion

Notwithstanding its long history, the MRD evaluation in FL and its significance in the clinical practice are still matter of debate. The recent European guidelines affirm that MRD has really got a predictive and prognostic significance, but that it is not still suitable for driving treatment of FL patients (Dreyling et al., 2017).

This is probably due to several considerations: (1) different studies proved that MRD-negative patients show a better prognosis, either when receiving chemo-immunotherapy or high-dose treatment. Nevertheless, these experiences have been conducted or in the context of large phase-III multicenter trials or, at the opposite, on small series of patients, and thus many physicians consider those results not fully exportable to their routine activity; (2) notwithstanding the standardization performed by some cooperative groups (van der Velden et al., 2003; Pott et al., 2013), the availability of a common, easily performable and cheap molecular method for investigating MRD is still lacking; (3) about MRD and R-bendamustine, few data are still available, and thus further information on this specific setting would be waited.

Our study is retrospective and involved a small number of patients, but it represents the picture of that occurs to FL patients in the real life. That statement is well supported by the observation that our clinical results are superimposable to those from other larger and pivotal trials: in particular, in our series CR rate resulted 79%, 2-years PFS 85%, and 2-years OS 94%. These values result comparable to those recently reported by Mondello et al. in 192 cases randomized to receive R-bendamustine or R-CHOP (Mondello et al., 2016). Those authors reported the superiority of R-bendamustine, with a significant prolongation of the median PFS (152 vs. 132 months); in that study, bendamustine offered 94% of ORR, and 63% of CR. Probably, the higher rate of overall response (100%) found in our series could be explained by the retrospective nature of this study (where enrolment was only based on the DNA availability).

Nevertheless, even with the limitation of the number of our cases, the present study could add some information about MRD in the setting of R-bendamustine to those previously reported by Zohren and colleagues in the contex of the StiL trial (Rummel et al., 2013).

Moreover, it is relevant to observe that our molecular data are perfectly in line with those previously reported by other authors: the *BCL2/JH* rearrangement at baseline has been detected in 62% of our patients; this is the same percentage reported in the Gadolin trial (Pott et al., 2015) and in the StiL study (Zohren et al., 2015), and slightly higher than that ourselves reported in previous FIL trials (52% and 51%) (Ladetto et al., 2013; Galimberti et al., 2014).

Also the number of cases with BM involvement documented by microscopic observation but without molecular marker at baseline was analogous to that previously reported (14% in the present study vs. 17% in the FOLL05 trial) (Galimberti et al., 2014): this phenomenon could be surely related to a possible poor BM sampling, but also to the emerging demonstration that at least 8–10% of the “PCR-negative” cases present some “rare” breakpoints in the *BCL2* gene (these rearrangements have not be evaluated in the present study).

Thus, our study would suggest that R-bendamustine exerts a so frequent and deep clearance of MRD that it would be able to by-pass the negative prognostic impact usually played by the MRD in other contexts, such as when R-CHOP, R-CVP, R-FM, or R-FND were adopted as induction regimens.

Indeed, in the present work the baseline molecular tumor burden did not condition the quality of response, opposite to that we observed in the FOLL05 study, where CR was achieved by the half of the initially PCR-positive in respect of the PCR-negative cases (Galimberti et al., 2014).

In the present study, relapsed patients presented an initial molecular burden 15 times higher than that measured in not relapsed/progressed cases, but this difference did not translate in a significant advantage in terms of PFS. This could be probably due to the small number of patients, but we cannot exclude that it could be also the consequence of the more rapid and deeper MRD clearance exerted by bendamustine: in our series, more than 90% of cases achieved the MRD negativity, and quantitative data showed that the majority of cases achieved a very deep reduction of the molecular disease. The median reduction of 4 logs is encouraging in respect of the 2 logs reported after R-CHOP (Galimberti et al., 2014) and the 2.5 logs reported after yttrium-ibritumomab tiuxetan treatment (Ibatici et al., 2014).

Interestingly, the rapidity of the molecular clearance offered by bendamustine has been already well demonstrated in the Gallium (Pott et al., 2016) and Gadolin (Pott et al., 2015) trials, where bendamustine was combined with obinutuzumab: in both trials, the rapid efficacy of the R-bendamustine also in molecular terms has been clearly demonstrated. It is also relevant that in the arm with R-bendamustine of the Gallium trial, 90% of patients became MRD-negative, a percentage comparable to that observed in our study (93%) and that in that trial, as in the present study, the MRD status did not condition the PFS length.

In conclusion, our study suggests that the combination of rituximab and bendamustine, by exerting a rapid and deep clearance of the molecular disease, could represent a highly effective therapeutic approach to FL, also in terms of molecular disease. The larger ongoing studies will show if the MRD would be used as adjunctive tool for better selecting the more effective treatment for FL patients.

## Ethics statement

This study was carried out in accordance with the Declaration of Helsinki. All patients gave the consent to leave the leftover of the routine analyses for scientific purposes. this statement was approved by the legal office of the AOUP.

## Author contributions

All authors participated to the conception of the study. EC, GE, SuGr, and FG performed PCR assays. SG, NC, MR, GC, FM, LI, FF, AB, LR, SK, DV, and MP enrolled patients, performed treatment and clinical follow-up. LM and EC performed the microscopic analyses. SG and MP performed statistical analyses. All authors approved the final version of the manuscript.

### Conflict of interest statement

The authors declare that the research was conducted in the absence of any commercial or financial relationships that could be construed as a potential conflict of interest.

## Abbreviations

FL: follicular lymphoma
BM: bone marrow
MRD: minimal residual disease
R: rituximab
PCR: polymerase chain reaction
PFS: progression-free survival
OS: overall survival
FIL: fondazione italiana linfomi
CR: complete remission
PR: partial remission
PET: positron-emission tomography
ESMO: European Society for Medical Oncology
ROC: receiving operating characteristics
FLIPI: follicular lymphoma international prognostic index.
